# Graphene-wrapped yolk–shell of silica-cobalt oxide as high-performing anode for lithium-ion batteries†

**DOI:** 10.1039/d4ra04236k

**Published:** 2024-09-23

**Authors:** Jingjing Ma, Jiawei Yong, Xiangnan Li, Huishuang Zhang, Yuanchao Li, Hongying Niu, Shuting Yang, Yu-Shi He, Zi-Feng Ma

**Affiliations:** a Postdoctoral Research Base, School of Chemistry and Chemical Engineering, Henan Normal University Xinxiang Henan 453007 PR China jingjingma_xx@163.com shutingyang@foxmail.com +86-0373-3040148; b Postdoctoral Station, School of Chemistry and Chemical Engineering, Henan Institute of Science and Technology Xinxiang Henan 453003 PR China; c Shanghai Electrochemical Energy Devices Research Center, School of Chemistry and Chemical Engineering, Shanghai Jiao Tong University Shanghai 200240 China

## Abstract

Silica (SiO_2_) shows promise as anode material for lithium-ion batteries due to its low cost, comparable lithium storage discharge potential and high theoretical capacity (approximately 1961 mA h g^−1^). However, it is plagued by issues of low electrochemical activity, low conductivity and severe volume expansion. To address these challenges, we initially coat SiO_2_ with CoO, followed by introducing SiO_2_@CoO into graphene sheets to fabricate an anode composite material (SiO_2_@CoO/GS) with uniformly dispersed particles and a 3D graphene wrapped yolk–shell structure. The coating of CoO on SiO_2_ converted the negative surface charge of SiO_2_ to positive, enabling effective electrostatic interactions between SiO_2_@CoO and graphene oxide sheets, which provided essential prerequisites for synthesizing composite materials with uniformly dispersed particles and good coating effects. Furthermore, the Co-metal formed during the charge–discharge process can act as a catalyst and electron transfer medium, activating the lithium storage activity of SiO_2_ and enhancing the conductivity of the electrode, conclusively achieving a higher lithium storage capacity. Ultimately, due to the activation of SiO_2_ by Co-metal during cycling and the excellent synergistic effect between SiO_2_@CoO and graphene, SiO_2_@CoO/GS delivers a high reversible capacity of 738 mA h g^−1^ after 500 cycles at 200 mA g^−1^. The product also demonstrates excellent rate performance with a reversible capacity of 206 mA h g^−1^ at a high specific current of 12.8 A g^−1^. The outstanding rate performance of SiO_2_@CoO/GS may be ascribed to the pseudo-capacitive contribution at high specific current upon cycling.

## Abstract 2

The SiO_2_ was first modified by CoO coating and then introduced into graphene to fabricate composites with high dispersibility.

## Graphical abstract

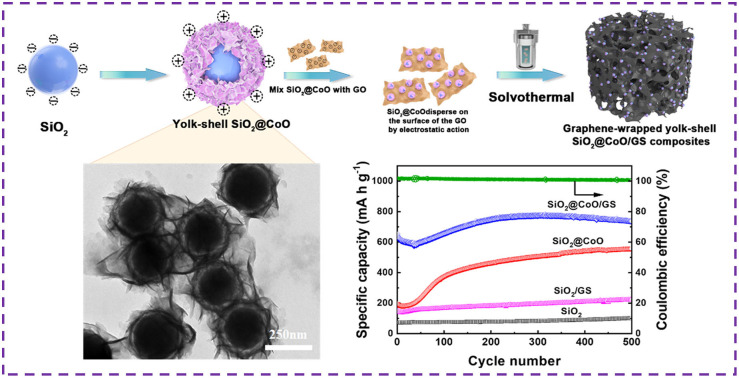

## 1 Introduction

Lithium-ion batteries (LIBs) have gained significant attraction in portable electronic devices and compact electric vehicles owing to their high energy density, long lifespan and absence of memory effect.^1^ Nonetheless, the prevailing energy density of commercial LIBs (∼250 W h kg^−1^) falls short of satisfying the heightened specific energy demands of electric vehicles and expansive energy storage installations.^2–5^ The primary approach to surmounting these challenges unquestionably revolves around the development of electrode materials with superior specific energy for advanced LIBs.

Silicon-based materials are considered as promising candidates to replace graphite for the next generation anode materials of LIBs due to their low lithium insertion potential, high capacity, high safety and abundant sources. Silicon anode materials, in particular, possess an ultra-high theoretical specific capacity of 4200 mA h g^−1^.^6^ However, they are costly to manufacture and have stringent production requirements.^7^ Additionally, during the lithiation process, silicon undergoes a volume expansion of more than 300% and significant mechanical stress, leading to particle fragmentation and continual breakdown and regeneration of the solid electrolyte interface (SEI), resulting in severe capacity degradation and poor rate performance.^8,9^ As a result, it is challenging to apply silicon in practical production processes. Although the theoretical specific capacity of SiO_2_ (1965 mA h g^−1^) is lower than that of silicon,^10^ it is still a viable alternative due to its simple preparation method, widespread availability and lower volume change during charge/discharge processes.

However, the performance of SiO_2_ in actual charge/discharge processes is limited by its high binding energy of Si–O bonds, making it difficult to fracture and activate SiO_2_, resulting in poor lithium storage reaction activity.^11^ Research has demonstrated that metals or metal oxides can catalyze and activate the lithium storage reactions of SiO_2_, thereby significantly enhancing its lithium storage capacity.^12^ Moreover, SiO_2_-based anodes suffer from low conductivity, slow lithium ion diffusion and severe volume expansion during charge/discharge processes.^13^ To resolve the issues, SiO_2_ can be designed and prepared into various nanostructured materials (such as nanoparticles, nanowires,^14,15^ nanorods, nanotubes,^16,17^ nanoporous structures,^18,19^*etc.*). This helps to mitigate the volume changes of SiO_2_ during reactions and shorten the paths for lithium ion and electron transport.^20,21^ Alternatively, superior carbon coating structures can be designed to further improve the volume expansion issues of SiO_2_ and enhance its conductivity.^22^

Graphene is a preferred carbon material to design composite due to its high conductivity, flexibility and strong malleability. By effectively designing methods and processes, combining SiO_2_ with graphene to create silica/graphene composites with graphene-wrapped structures can effectively mitigate the volume effects of SiO_2_ and enhance material conductivity, thereby significantly enhancing the electrochemical performance of silica-based anodes.^22–24^ The dispersion of particles, graphene wrapping and pore size distribution in graphene/silica-based composites significantly impact their electrochemical performance. However, the high specific surface energy causes significant agglomeration of nano-SiO_2_ in composites. Furthermore, the electronegative nature of both SiO_2_ and graphene oxide (GO) surfaces presents a challenge in fabricating nano-SiO_2_/graphene composites with effective graphene-wrapped structures and highly dispersed SiO_2_ using simple methods.^25,26^ To address this obstacle, it is necessary to alter the surface charge properties of SiO_2_ to establish effective electrostatic interactions between SiO_2_ and GO.^24^

Based on this, this study proposes a novel preparation method by first modifying the surface of SiO_2_ with metal oxide coating to impart positive charge and then combining it with negatively charged graphene oxide. In colloid science,^27^ metal oxides dispersed in media tend to selectively adsorb cations and exhibit positive charge,^28,29^ contrary to the electronegative surfaces of graphene oxide. Thus, SiO_2_ modified with metal oxide coating (SiO_2_@CoO) can initially disperse and adsorb on the surface of graphene oxide through electrostatic interactions, ultimately obtaining uniformly dispersed silica particles individually wrapped by graphene. This effectively controls the volume expansion of SiO_2_ during lithium insertion/extraction and enhances the conductivity of electrode. Furthermore, CoO not only participates in lithium storage reactions but also the Co metal formed during charge/discharge processes can act as a catalyst and electron transfer medium, activating the lithium storage activity of SiO_2_ and enhancing its conductivity, thereby achieving a higher lithium storage capacity. The study ultimately produced a composite material, SiO_2_@CoO/GS, with a 3D graphene wrapped yolk–shell structure, exhibiting a capacity of 738 mA h g^−1^ after 500 cycles at a current density of 200 mA g^−1^. Additionally, it demonstrates excellent rate performance, retaining a specific capacity of 206 mA h g^−1^ at a high current density of 12.8 A g^−1^.

## 2 Experimental section

### 2.1 Synthesis of SiO_2_@CoO composite

SiO_2_ was prepared using the Stöber solution-gelation process and see the detailed process in the ESI.†

A mixture containing Co(NO_3_)_2_·6H_2_O (0.8 g), cetyltrimethylammonium bromide (CTAB) (0.2 g), SiO_2_ (0.8 g) and isopropanol (6 mL) was mixed with 24 mL of deionized water using magnetic stirring for 2 hours to create a homogeneous suspension. The mixture was then transferred to a Teflon-lined autoclave and maintained at 180 °C for 20 hours. Following the cooling process to room temperature (20–25 °C), the SiO_2_@CoO precursor was obtained following washing and drying.

### 2.2 Synthesis of SiO_2_@CoO/GS and SiO_2_/GS composites

GO was prepared using a modified Hummers' method, which was described in detail in the ESI.† GO (40 mg) and SiO_2_@CoO (80 mg, 300 nm) were mixed in 40 mL of absolute ethanol by magnetic stirring for 2 hours and sonication lasting 1 hour to create a uniform suspension. The solution was then placed in a Teflon-lined autoclave and kept at 200 °C for 12 hours. The resulting sample was washed with deionized water to remove ethanol before undergoing freeze-drying to obtain the SiO_2_@CoO/GS composite.

For comparison, SiO_2_/GS was directly synthesized through a solvothermal process following the thorough dispersion of graphene and SiO_2_ nanoparticles in an absolute ethanol suspension.

### 2.3 Material characterization

X-ray diffraction (XRD) patterns were acquired on a Bruker X-ray diffractometer (D8 Advance A25) with Cu-K_α_ radiation. The surface area was determined by a nitrogen adsorption/desorption analyzer (Micromeritics ASAP2460) and Brunauer–Emmett–Teller (BET) method. Fourier transform infrared spectra (FTIR) were obtained by a TENSOR 27 instrument. X-ray photoelectron spectroscopy (XPS) was employed to investigate the surface chemistry of the samples using a Kratos Axis Ultra DLD spectrometer. Transmission electron microscopy (TEM) and scanning TEM (STEM) analyses were conducted using a JEM-2100F instrument with integrated energy-dispersive X-ray spectroscopy (EDS). The contents of Si and Co elements in samples were determined by an inductively coupled plasma optical emission spectrometer (ICP-OES, 725 ES).

### 2.4 Electrochemical measurements

The working electrode slurry was prepared by blending active materials, Super-P and polyacrylic acid (PAA) with mass ratio of 6 : 2 : 2 using *N*-methyl-2-pyrrolidinone (NMP) as solvent. The resulting slurry was applied onto copper foil, dried in vacuum at 60 °C for 12 hours and assembled to form the working electrode with an active material loading of approximately 1.2 mg cm^−2^. Coin cells (CR2016) were assembled in an argon-filled glove box using Li foil as the counter electrode, a microporous polyethylene membrane as the separator and 1.0 mol L^−1^ LiPF_6_ in a mixture of ethylene carbonate (EC) and dimethyl carbonate (DMC) (v/v, 1 : 1) with 10 vol% fluoroethylene carbonate (FEC) as electrolyte.

Electrochemical experiments for the half-cells were performed using battery test system (NEWARE BTS7.6.0) within voltage window of 0.005–3 V (*vs.* Li/Li^+^) at room temperature. Charge/discharge capacities were normalized based on the weight of active materials in the electrodes. Cyclic voltammetry (CV) measurements were conducted using a CHI 604E electrochemical workstation (Shanghai Chenhua Instrument Co.) at scan rate of 0.1 mV s^−1^. Electrochemical impedance spectroscopy (EIS) was carried out using the same workstation over a frequency range spanning from 100 kHz to 0.1 Hz.

## 3 Discussion

**Fig. 1:**
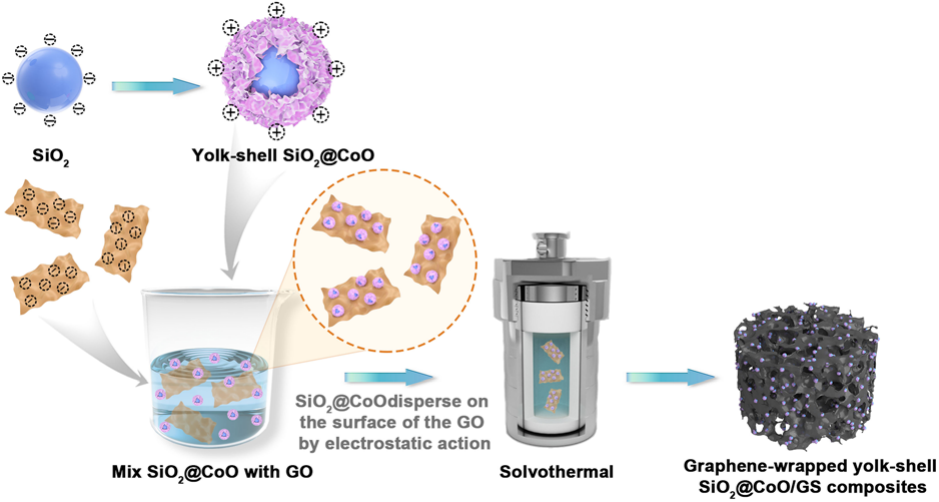
The preparation process of SiO_2_@CoO/GS composite.

The preparation process of the SiO_2_@CoO/GS composite material is illustrated in Fig. 1 In the first step, SiO_2_, CTAB and Co(NO_3_)_2_·6H_2_O were mixed in a mass ratio of 4 : 1 : 4. Through hydrothermal reaction and the action of CTAB, sheet-like CoO grew and crystallized on the surface of SiO_2_, forming the precursor SiO_2_@CoO with a yolk–shell structure. With the modification of CoO, the surface of SiO_2_ particles shifted from electronegative to electropositive. In the second step, SiO_2_@CoO was mixed with graphene oxide (GO) in a mass ratio of 2 : 1, allowing the electropositive SiO_2_@CoO particles to be uniformly dispersed and adsorbed onto the electronegative surface of GO through electrostatic attraction. Subsequently, during solvothermal reaction, GO was gradually reduced to graphene (GS). Through π–π bonding, GS contracted and cross-linked to form a three-dimensional porous network structure, encapsulating SiO_2_@CoO particles, ultimately producing the 3D graphene-wrapped SiO_2_@CoO composite material SiO_2_@CoO/GS.

**Fig. 2:**
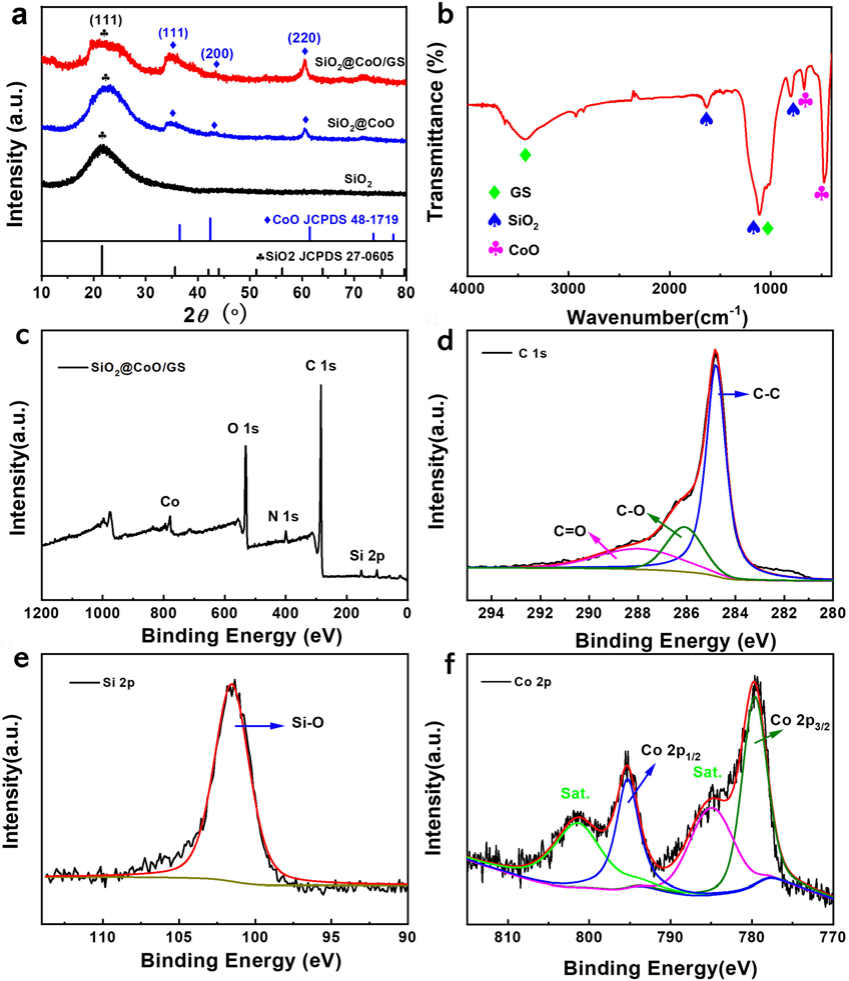
(a) XRD patterns of SiO_2_, SiO_2_@CoO and SiO_2_@CoO/GS; (b) FT-IR spectrum of SiO_2_@CoO/GS; (c) XPS spectrum of SiO_2_@CoO/GS composite; (d) C 1s, (e) Si 2p and (f) Co 2p XPS spectra.

The synthesized samples were firstly analyzed by XRD, as shown in Fig. 2a. The self-made SiO_2_ exhibits a broad peak at 22°, matching well with the standard peak of SiO_2_ (JCPDS 27-0605), corresponding to the (111) crystal plane of amorphous SiO_2_. Both SiO_2_@CoO and SiO_2_@CoO/GS show diffraction peaks at 36°, 42° and 62°, which are attributed to the planes of (111), (200) and (220) of CoO (JCPDS 48-1719), respectively. No characteristic peaks of graphene oxide or graphene can be observed in the SiO_2_@CoO/GS composite at 11° and 22–28°,^27^ indicating the successful reduction of graphene oxide during the solvent thermal process and the prevention of interlayer stacking of graphene by embedding SiO_2_@CoO particles.^30^

Fig. 2b depicts the FTIR spectrum of SiO_2_@CoO/GS. The strong peak at 476 cm^−1^ is attributed to the combined action of Si–O and Co–O bonds. The peak at 675 cm^−1^ can be attributed to the stretching vibration of Co–O bonds.^31^ The peak at 804 cm^−1^ is associated with Si–O bond vibrations. The peak at 1112 cm^−1^ corresponds to the anti-symmetric stretching vibration of Si–O–Si and C–O bonds in graphene, while the broad peak at 3430 cm^−1^ represents the bending vibration of the –OH group in graphene.^32,33^

X-ray photoelectron spectroscopy (XPS) analysis was further conducted. As shown in Fig. 2c, the elements Co, O, C and Si were detected. In the C 1s spectrum (Fig. 2d), the peaks at 284.8 eV, 286.2 eV and 288 eV correspond to C–C, C–O and C=O bonds, respectively.^34^ The high intensity of the C–C bond in C 1s indicates that the GO in SiO_2_@CoO/GS was reduced to graphene. The peak at 101.7 eV in the Si 2p_3/2_ spectrum (Fig. 2e) is attributed to the characteristic peak of Si^4+^.^19,20^ The Co 2p spectrum (Fig. 2f) shows two representative peaks at 795.7 eV and 780.4 eV, corresponding to Co 2p_1/2_ and Co 2p_3/2_ of Co^2+^.^17,18^ The results of XPS analysis are consistent with XRD and FT-IR, confirming the successful synthesis of SiO_2_@CoO/GS. As shown in Fig. S1,† the N 1s absorption peak at 401.35 eV corresponds to the C–NH and (–N + (CH_3_)_2_–/–N + (CH_3_)_3_) functional groups derived from CTAB.^35,36^ The surface characteristics of SiO_2_@CoO may be modified by these functional groups through electrostatic interactions or chemical bonding, which could enhance its interaction with GO and potentially improve the material's electrochemical performance.^37,38^

To further determine the proportion of each component in SiO_2_@CoO/GS samples, ICP-OES was employed. As displayed in Table S1,† the mass percentage (wt%) of Si and Co in SiO_2_@CoO/GS are 28% and 13.954%, respectively. According to that, the content of SiO_2_, CoO and GS in SiO_2_@CoO/GS can be calculated to be 60 wt%, 17.7 wt%, 22.3 wt%, respectively.

**Fig. 3:**
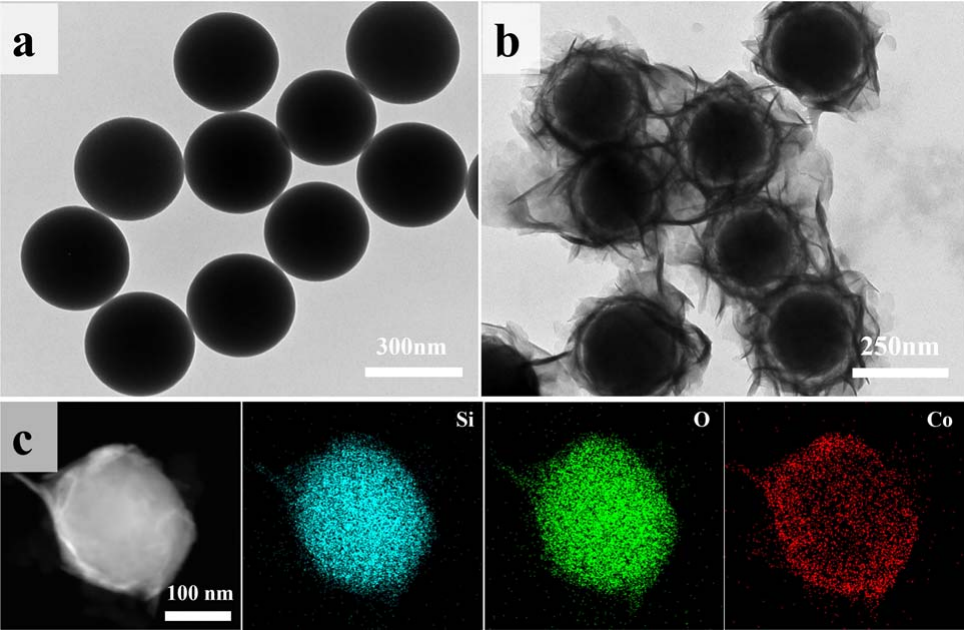
TEM images of (a) SiO_2_ and (b) SiO_2_@CoO; (c) elemental mapping of SiO_2_@CoO.

Microscopic morphology and elemental distribution of the materials were analyzed using SEM and TEM. Fig. 3a and S2(a)† show the TEM and SEM images of the self-made SiO_2_, revealing smooth and uniformly sized (∼250 nm) spherical particles. After CoO encapsulation, the surface smoothness of the particles decreased (Fig. S2b†). In Fig. 3b, ring-shaped gaps between CoO and SiO_2_ are clearly observed, indicating the growth of lamellar CoO on the surface of SiO_2_, eventually forming a yolk–shell structure. Fig. 3c presents the STEM elemental mapping of SiO_2_@CoO, showing uniform distribution of Si, Co and O elements on the surface of SiO_2_, indicating successful preparation of CoO-coated SiO_2_ material.

Table 1 presents the zeta potentials for pure SiO_2_, SiO_2_@CoO and GO. It can be observed that the surface of self-made SiO_2_ exhibits negative charge (−5.69), which changes to +214.53 after being encapsulated by CoO. The coating structure of CoO not only successfully changes the surface charge of SiO_2_ from negative to positive, but also provides sufficient buffer space for the volume effect of SiO_2_ through the formation of ring-shaped gaps. Furthermore, the ring-shaped gaps also facilities the insertion and extraction of lithium ions.

**Table 1:** Zeta potential of SiO_2_, SiO_2_@CoO and GO

Samples	SiO_2_	SiO_2_@CoO	GS
Zeta potential (mV)	−5.69	214.53	−44.5 ± 9.08

**Fig. 4:**
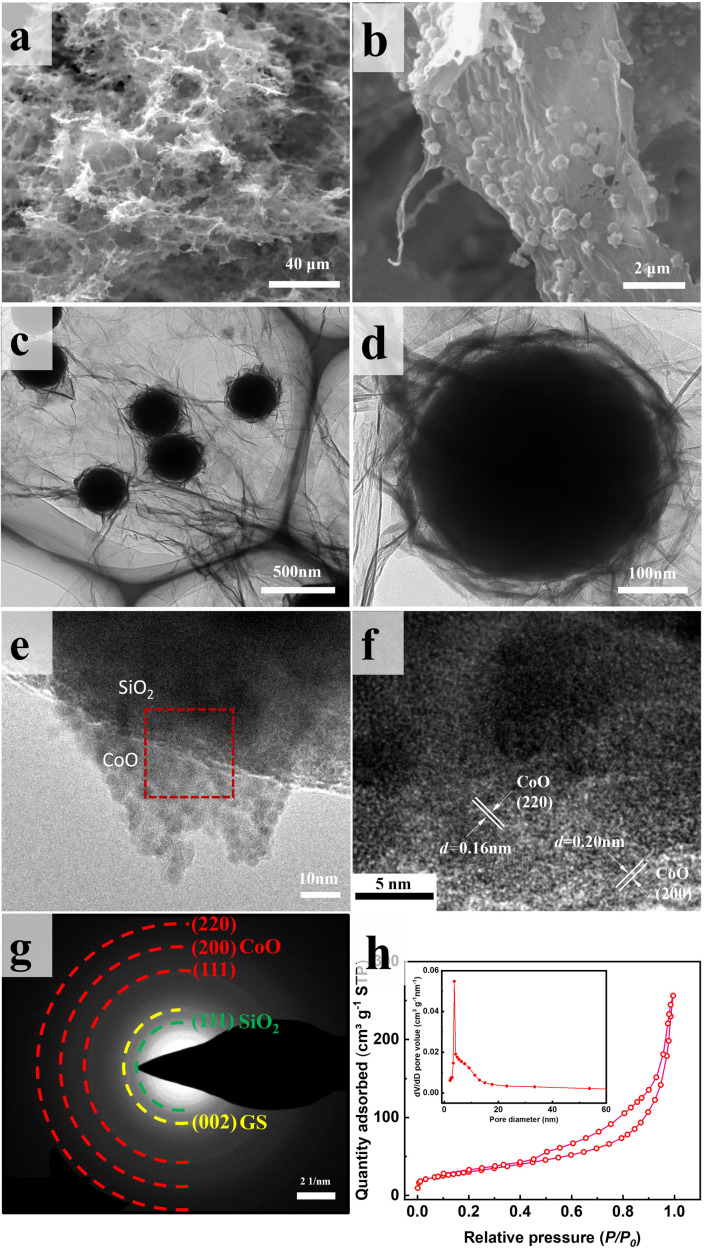
(a and b) SEM images, (c–e) TEM images, (f) HRTEM image, (g) SAED pattern and (h) BET image of SiO_2_@CoO/GS.

Fig. 4 displays SEM and TEM images of SiO_2_@CoO/GS. The graphene sheets form a three-dimensional porous network structure through interlayer cross-linking, proving multi-dimensional channels for rapid electrons and lithium ions transport (Fig. 4a). The CoO-coated SiO_2_ particles (SiO_2_@CoO) with a yolk–shell structure are uniformly distributed between the graphene layers and completely enveloped by graphene (Fig. 4b–d). Fig. 4e displays the sheet-like CoO on the surface of SiO_2_ in SiO_2_@CoO/GS. The diffraction stripes in Fig. 4f belong to the 200 and 220 crystal planes of CoO. Fig. 4g presents regular diffraction rings attributed to CoO (220, 200 and 110 crystal planes), SiO_2_ (111 crystal plane) and GS (002 crystal plane).

Fig. S3† presents the EDS mapping images of SiO_2_@CoO/GS, showing the uniform distribution of carbon elements on the surface of SiO_2_@CoO, indicating the successful preparation of composite with graphene cladding. The pleated graphene cladding not only enhances interface electrical contact but also prevents particle agglomeration and provides effective cushioning space to alleviate stress and strain induced by volume changes in the electrode material during cycling.^24^

As a comparison, SiO_2_/GS was also prepared using self-made SiO_2_ and GO as raw materials *via* a similar method. As shown in Fig. S4,† SiO_2_ particles were completely agglomerated and do not form a graphene cladding structure. This clearly demonstrates that the surface coating of CoO on SiO_2_ plays a crucial role in modifying its surface properties and in the synthesis of composite materials with excellent structural characteristics.

The N_2_ adsorption–desorption isotherm of SiO_2_@CoO/GS is presented in Fig. 4h. The presence of mesoporous structures is indicated by the obvious hysteresis in the high relative pressure region.^39^ The Barrett–Joyner–Halenda (BJH) pore size of these pores ranges from approximately 2 to 10 nm (insert in Fig. 4h), mainly originating from the mesoporous structures present in the ring-shaped gaps between CoO and SiO_2_. Furthermore, a BET surface area of 107.86 m^2^ g^−1^ and a cumulative pore volume of 0.40 cm^3^ g^−1^ for SiO_2_@CoO/GS were determined. The mesoporous structure of SiO_2_@CoO/GS facilitates ions transfer and provides sufficient buffer space for the volume changes of SiO_2_.

**Fig. 5:**
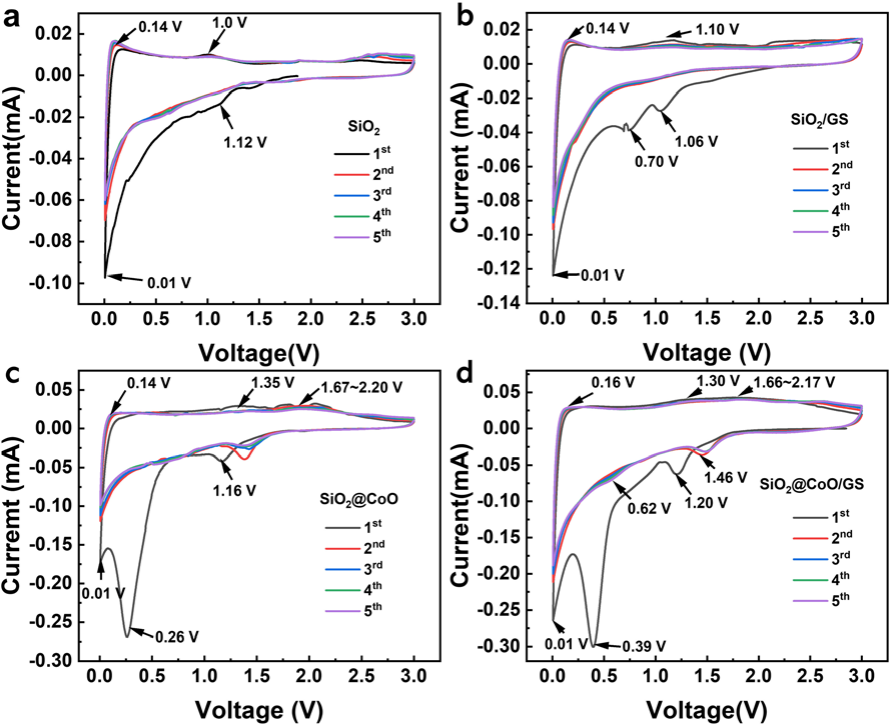
CV curves of the SiO_2_ (a), SiO_2_/GS (b), SiO_2_@CoO (c) and SiO_2_@CoO/GS (d) at 0.1 mV s^−1^.

Fig. 5 presents the cyclic voltammetry (CV) curves of SiO_2_, SiO_2_@CoO, SiO_2_/GS and SiO_2_@CoO/GS at a scanning rate of 0.1 mV s^−1^. According to previous studies, the electrochemical reactions of CoO, SiO_2_ and GS with lithium can be described as follows:^12,24,40–42^

1 SiO_2_ + 4Li^+^ + 4e^−^ → 2Li_2_O + Si

2 5SiO_2_ + 4Li^+^ + 4e^−^ ↔ 2Li_2_Si_2_O_5_ + Si

3 2SiO_2_ + 4Li^+^ + 4e^−^ → Li_4_SiO_4_ + Si

4 Si + *x*Li^+^ + *x*e^−^ ↔ Li_*x*_Si

5 CoO + 2Li^+^ + 2e^−^ ↔ Co + Li_2_O

6 6C + Li^+^ + e^−^ ↔ LiC_6_

For pure SiO_2_, the peak at 1.12 V is associated with the reduction of amorphous SiO_2_ to Si and the formation of Li_2_O, Li_4_SiO_4_ and Li_2_Si_2_O_5_ (eqn (1)–(3)). A weak peak observed at ∼0.01 V corresponds to the alloy reaction of Si (eqn (4)). In the charge process, a small peak at ∼0.14 V corresponds to the de-alloying of Li–Si and an oxidation peak around 1.0 V may be attributed to the reversible conversion reaction between Li_2_Si_2_O_5_ and SiO_2_ (eqn (2)).

SiO_2_/GS (Fig. 5b) exhibits similar oxidation-reduction peak positions to pure SiO_2_, with an additional reduction peak around 0.70 V attributed to the formation of a solid-electrolyte interphase (SEI) film on the surface of the graphene.

SiO_2_@CoO/GS (Fig. 5d) and SiO_2_@CoO (Fig. 5c) display similar peak positions. The reduction peak at ∼1.20 V in the first scan corresponds to the conversion reaction between SiO_2_ and Si (eqn (1)–(3)). From the second cycle onwards, the irreversible reactions disappear and the peak at 1.20 V shifts to around 0.62 V, corresponding to the reversible reaction in eqn (2). The peak at 0.39 V is attributed to the reduction of CoO to Co and the formation of the SEI film.^12,41,43,44^ This peak disappears in subsequent cycles and the conversion peak from CoO to Co shifts to 1.46 V.^24,45^ The peak around 0.01 V corresponds to the alloy reaction of Si. During charging, the weak peak at 0.16 V corresponds to the de-alloying of Li–Si, while the peak at 1.30 V corresponds to the reversible conversion reaction between Li_2_Si_2_O_5_ and SiO_2_. The peaks from 1.66 to 2.17 V indicate the oxidation of Co metal during de-lithiation.^24,44^ Compared with SiO_2_ and SiO_2_/GS, the intensity of the main reduction peak (∼0.01 V) corresponding to the lithiation reactions of Si in SiO_2_@CoO and SiO_2_@CoO/GS are significantly higher and their integrated area of the cyclic voltammetry curves are larger (Fig S5†), indicating that the materials with CoO have higher reactivity and lithium storage capacity. This may be related to the catalysis effect of Co metal which reduced from CoO. The generated Co not only activate SiO_2_ by breaking the Si–O bonds, thereby promoting the conversion reaction of SiO_2_ to Si, but also catalyze the lithiation reaction of Si.^11,24,45^ In addition, Co metal can also collaborate with graphene to provide fast electron transfer channels for materials, thereby further enhancing their lithium storage performance.

**Fig. 6:**
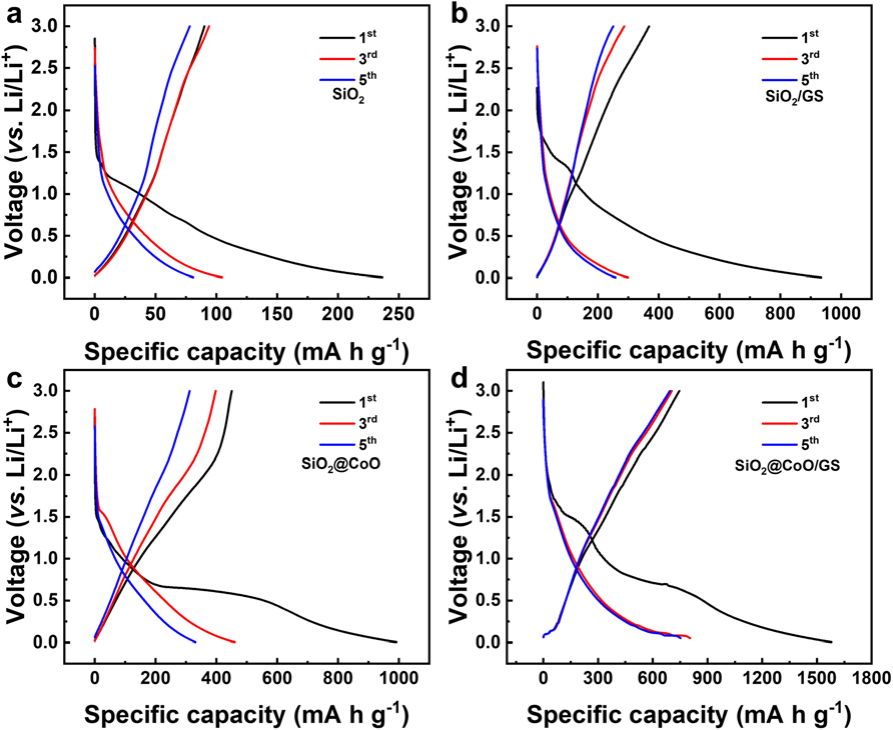
The charge/discharge voltage profiles of pure SiO_2_ (a), SiO_2_/GS (b), SiO_2_@CoO (c) and SiO_2_@CoO/GS (d) for the 1st, 3rd and 5th cycles at a current density of 50 mA g^−1^ in the voltage range of 0.005 to 3 V.

Fig. 6 shows the charge/discharge voltage profiles of pure SiO_2_, SiO_2_/GS, SiO_2_@CoO and SiO_2_@CoO/GS for the 1st, 3rd and 5th cycles at a current density of 50 mA g^−1^. The slopes and plateaus in the charge/discharge of these materials correspond closely to the peak positions in respective CV curves. In the first discharge process of SiO_2_@CoO/GS (Fig. 6d), the discharge plateau around 1.48 V corresponds to the lithiation reaction of SiO_2_, while the plateau around ∼0.6 V mainly due to the lithiation of CoO and the formation of the SEI film. The irreversible phases formed during the first cycle, such as Li_2_O and Li_4_SiO_4_, along with the generation of the SEI film, consume significant amount of lithium, leading to the low initial coulomb efficiency.^46,47^ The plateau around ∼0.1 V in the charge process is mainly attributed the conversion reaction of Si to Li_*x*_Si, which is the main source of reversible capacity. The first discharge capacity of SiO_2_@CoO/GS can reach 1579 mA h g^−1^ with a charge capacity of 746 mA h g^−1^ and coulombic efficiency of 47.2%. The first discharge/charge capacities/coulombic efficiencies of SiO_2_ (Fig. 6a), SiO_2_/GS (Fig. 6b) and SiO_2_@CoO (Fig. 6c) are 236 mA h g^−1^/89 mA h g^−1^/37%, 933 mA h g^−1^/368 mA h g^−1^/39.4% and 992 mA h g^−1^/449 mA h g^−1^/45.2%, respectively.

It can be concluded that SiO_2_@CoO/GS exhibits higher capacity and coulombic efficiency, mainly due to two reasons: (1) catalytic activation effect of Co metal on SiO_2_ effectively increases the revers of the reactions, enhancing the coulombic efficiency; (2) the graphene-coated structure enhances the dispersion and conductivity of the material, increases the effective active surface area and thus improves the storage capacity of lithium.

**Fig. 7:**
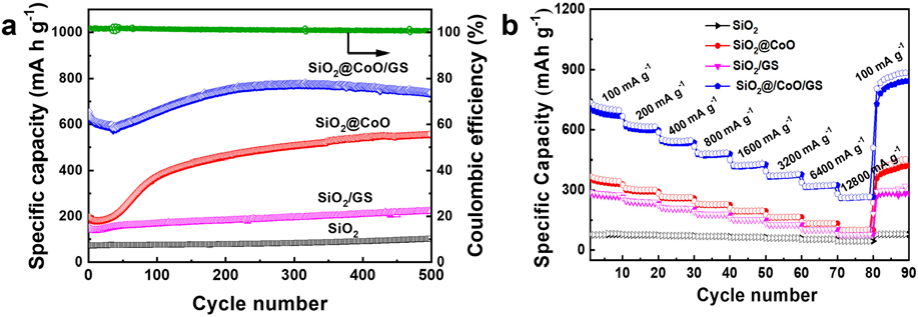
(a) Cycling performance of SiO_2_, SiO_2_/GS, SiO_2_@CoO and SiO_2_@CoO/GS electrodes measured at 200 mA g^−1^; (b) rate capability of SiO_2_, SiO_2_/GS, SiO_2_@CoO and SiO_2_@CoO/GS electrodes at 100–12800 mA g^−1^.

Fig. 7a illustrates the cycling performance of SiO_2_@CoO/GS, SiO_2_@CoO, SiO_2_/GS and SiO_2_. All cells underwent an activation process at 50 mA g^−1^ for 5 cycles before each test. SiO_2_@CoO/GS demonstrates the best cycling stability among the electrode materials, maintaining a specific capacity of 738 mA h g^−1^ after 500 cycles at a current density of 200 mA g^−1^, far surpassing SiO_2_@CoO (558 mA h g^−1^), SiO_2_/GS (223 mA h g^−1^) and SiO_2_ (103 mA h g^−1^). The capacity increase observed with cycling for all four materials is a common phenomenon in silicon-based materials, attributed to the activation process and gradual pulverization of larger particles into smaller ones during cycling.^12,26,42,47,48^ A notable observation is the significant capacity increase for SiO_2_@CoO/GS and SiO_2_@CoO further confirm the catalytic activation effect of the Co metal on SiO_2_. Compared to SiO_2_@CoO, SiO_2_@CoO/GS has a smoother capacity increase curve, which is mainly attributed to the graphene-wrapped structure. It effectively suppresses the excessive expansion of SiO_2_@CoO particles, resulting in more stable cycling performance.

To assess electrode kinetics, the rate capabilities of SiO_2_@CoO/GS, SiO_2_@CoO, SiO_2_/GS and SiO_2_ anodes were examined at different current densities ranging from 100 to 12 800 mA g^−1^ in Fig. 7b. All cells were activated at 50 mA g^−1^ for 5 cycles prior to the rate tests. The SiO_2_@CoO/GS anode exhibits discharge capacities of 707, 617, 543, 480, 426, 374, 322 and 264 mA h g^−1^ at current densities of 100, 200, 400, 800, 1600, 3200, 6400 and 12 800 mA g^−1^, respectively. Furthermore, upon reverting the current density back to 100 mA g^−1^, the reversible capacity can recover to 866 mA h g^−1^, showcasing the exceptional rate capability of the SiO_2_@CoO/GS anode. The cycling and rate performance of the SiO_2_@CoO/GS synthesized in this work have been compared with those of other silicon-based composites reported in the literature, and the results are summarized in Table S2.† Compared to other silicon-based composites, the SiO_2_@CoO/GS prepared in this study demonstrates superior cycling stability and rate capability.

**Fig. 8:**
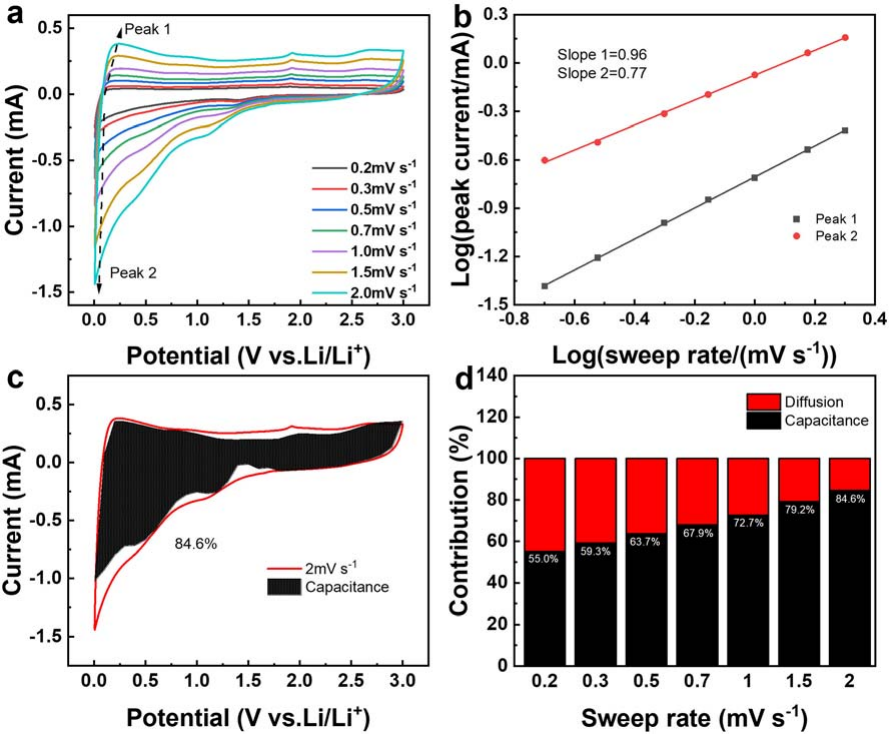
(a) The CV curves of SiO_2_@CoO/GS at 0.2, 0.3,0.5, 0.7,1.0, 1.5 and 2.0 mV s^−1^; (b) relationships between peak currents and sweep rates for determining the *b* values of the anodic and cathodic peaks for SiO_2_@CoO/GS; (c) the CV curve of SiO_2_@CoO/GS at 2 mV s^−1^ with an estimated capacitive contribution in the shaded region; (d) normalized contribution ratios of capacitive and diffusion-controlled capacities of SiO_2_@CoO/GS at various scan rates from 0.2 to 2 mV s^−1^.

Fig. 8a presents the cyclic voltammetry (CV) curves of SiO_2_@CoO/GS at scan rates of 0.2, 0.3, 0.5, 0.7, 1.0, 1.5 and 2.0 mV s^−1^. Based on the equation of *I*_p_ = *av*^*b*^,^49^ the correlation between peak current (*I*_p_) and scan rate (*v*) is determined to ascertain the *b* value for the anodic and cathodic peaks of SiO_2_@CoO/GS, as shown in Fig. 8b. The slope *b* for the anode and cathode peaks of the SiO_2_@CoO/GS composite are found to be 0.96 and 0.77, respectively, indicating the coexistence of diffusion-controlled and capacitance processes.^50^ The ratio of capacitive contribution to diffusion-controlled contribution can be calculated using the equation *I* = *K*_1_*v* + *K*_2_*v*^1/2^.^49^Fig. 8c demonstrates that the capacitive-dominated contribution rate reaches 84.6% for the SiO_2_@CoO/GS composite at a scan rate of 2.0 mV s^−1^. Furthermore, the capacitive-dominated rate of SiO_2_@CoO/GS increases with the scan rate ranging from 0.2 to 2.0 mV s^−1^, as illustrated in Fig. 8d. The exceptional rate performance of SiO_2_@CoO/GS can be attributed to the pseudo capacitance-dominated storage mechanism.^34,51^ The presence of this mechanism contributes significantly to the battery's outstanding rate capability.

The electrodes after 40 cycles were further investigated using TEM to explore the structural stability of SiO_2_@CoO/GS. As shown in Fig. S6a and b,† the particle size of pure SiO_2_ sphere and SiO_2_ in SiO_2_/GS composite show little changes, indicating low reactivity of SiO_2_ with Li^+^ ions. Only a small percentage of surface SiO_2_ participates in reactions without activation, resulting in very low capacity. In contrast, SiO_2_@CoO electrode material exhibits significant volume changes after cycling due to the catalytic and activation effects of CoO, leading to more SiO_2_ participating in lithiation/delithiation reactions and causing larger volume changes. When SiO_2_@CoO is further coated with graphene sheets (GS), the volume changes of the particles are effectively controlled, benefiting from the encapsulation effect of GS. This is the primary reason why SiO_2_@CoO/GS exhibits relatively stable cycling performance when compared to SiO_2_@CoO material.

## 4 Conclusion

In summary, SiO_2_@CoO/GS with a 3D cross-linked graphene-wrapped yolk–shell structure was successfully fabricated by implementing surface modification and a solvothermal electrostatic self-assembly process. Coating CoO onto the surface of SiO_2_ serves two main purposes: Firstly, it modifies the negatively charged SiO_2_ surface to a positively charged one, establishing effective electrostatic interactions between SiO_2_@CoO and GO for the preparation of composites with uniformly dispersed particles and well-formed graphene-encapsulated structure. Secondly, the Co metal formed during charge/discharge processes can act as a catalyst and electron transfer mediator, positively affecting the lithiation activity of SiO_2_ and enhancing its conductivity, thus improving the lithium storage capacity of SiO_2_. Subsequently, through the solvothermal process, positively modified SiO_2_@CoO particles are introduced into the 3D graphene, resulting in an anode material, SiO_2_@CoO/GS, with uniform particle dispersion and a 3D cross-linked graphene-wrapped yolk–shell structure. The 3D network structure of graphene provides multiple transfer channels for electrons and ions, while the graphene-wrapped yolk–shell structure effectively mitigates the volume effects of SiO_2_. Therefore, under the dual effects of Co catalytic activation and graphene-encapsulated structure, the SiO_2_@CoO/GS composite exhibits excellent electrochemical performance, with an initial discharge capacity of up to 1579 mA h g^−1^ and a specific capacity of 739 mA h g^−1^ after approximately 500 cycles at a current density of 200 mA g^−1^. Additionally, it demonstrates outstanding rate capability, maintaining a capacity of 206 mA h g^−1^ at a high current density of 12.8 A g^−1^.

## Data availability

The original contributions presented in the study are included in the article/ESI;† further inquiries can be directed to the corresponding author/s.

## Author contributions

J. J. Ma and S. T. Yang conceived the project. J. J. Ma and J. W. Yong designed the research scheme. J. W. Yong conducted the material synthesis, electrochemical tests, and material characterization. J. J. Ma and J. W. Yong wrote the original manuscript and analyzed most of the experimental data with the help of H. Y. Niu, Y. C. Li, and H. S. Zhang. X. N. Li performed the STEM test. S. T. Yang, Y. S. He, and Z. F. Ma reviewed the manuscript and provided the major revisions. All authors discussed the results and commented on the manuscript.

## Conflicts of interest

There are no conflicts to declare.

## Supplementary Material

RA-014-D4RA04236K-s001
