# Supplementary material for: Graphene-wrapped yolk–shell of silica-cobalt oxide as high-performing anode for lithium-ion batteries
Source: RSC Adv. 2024 Sep 23;14(41):30102–9. doi: 10.1039/d4ra04236k (PMC11417458; doi:10.1039/d4ra04236k)
Supplement: RA-014-D4RA04236K-s001 [file RA-014-D4RA04236K-s001.pdf]

Supplementary Information (SI) for RSC Advances.
This journal is © The Royal Society of Chemistry 2024

Supporting Information

Graphene-wrapped yolk-shell of silica-cobalt oxide as high-performing anode for lithium-ion batteries

Jingjing Ma,[a,b] Jiawei Yong,[b] Xiangnan Li,[a] Huishuang Zhang,[a] Yuanchao Li,[b] Hongying Niu,[b] Shuting Yang,*[a] Yu-Shi He[c] and Zi-Feng Ma[c]*

a. Postdoctoral Research Base, School of Chemistry and Chemical Engineering, Henan Normal University, Xinxiang, Henan 453007, PR China. E-mail: jingjingma_xx@163.com; shutingyang@foxmail.com; Fax: +86-0373-3040148

b. Postdoctoral Station, School of Chemistry and Chemical Engineering, Henan Institute of Science and Technology, Xinxiang, Henan 453003, PR China

c. Shanghai Electrochemical Energy Devices Research Center, School of Chemistry and Chemical Engineering, Shanghai Jiao Tong University, Shanghai 200240, China

* Corresponding author: jingjingma_xx@163.com; shutingyang@foxmail.com

EXPERIMENTAL SECTION

Preparation of SiO_2: SiO_2 was prepared via a Stöber sol-gel process. Aqueous ammonia (13 mL) and isopropanol (63.3 mL) were firstly added to 23.5 mL deionized water to form a uniform mixed suspension. Then added 5 mL of tetraethyl orthosilicate to the mixed suspension and stirred violently for 2 hours. After repeated centrifugation and sufficient drying, SiO_2 (300 nm) was finally obtained.

Preparation of GO: GO was synthesized by a modified Hummers' method. First, potassium peroxydisulfate (2.5 g), phosphoric anhydride (2.5 g) and flake graphite (5 g) were added to 50 mL of concentrated sulfuric acid (with a mass fraction of 98%) to form a mixed solution. Then the mixed solution was heated to 80 °C and stirred at a constant temperature for 6 h to complete the pre oxidation process. The pre-oxidized graphite was then washed and dried repeatedly. The pre-oxidized graphite solid and 15 g of potassium permanganate solid were successively added to 115 ml of concentrated sulfuric acid under ice water bath, fully stirred for 3.5 hours, then slowly added 400 ml of ultrapure water, stirred for 2 hours, and then added 20 ml of H_2O_2 (with a mass fraction of 30%). After repeated centrifugation, washing and dialysis treatment, GO was finally obtained after drying.

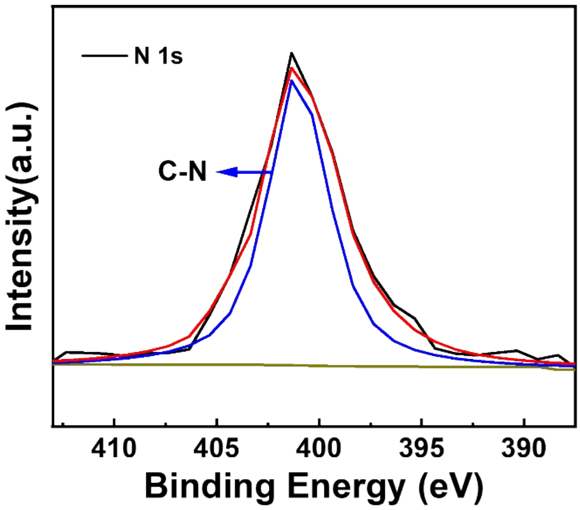

Fig. S1 N 1s XPS spectra.

Table S1 The ICP-OES results of SiO_2@CoO/GS.

Sample	Element	Weight (mg)	Volume (mL)	Dilution factor	Instrument reading (mg L^{-1})	Concentration (wt.%)
SiO_2@CoO/GS	Si	1.4	50	1	3.91	28.0%
	Co				7.84	13.954%

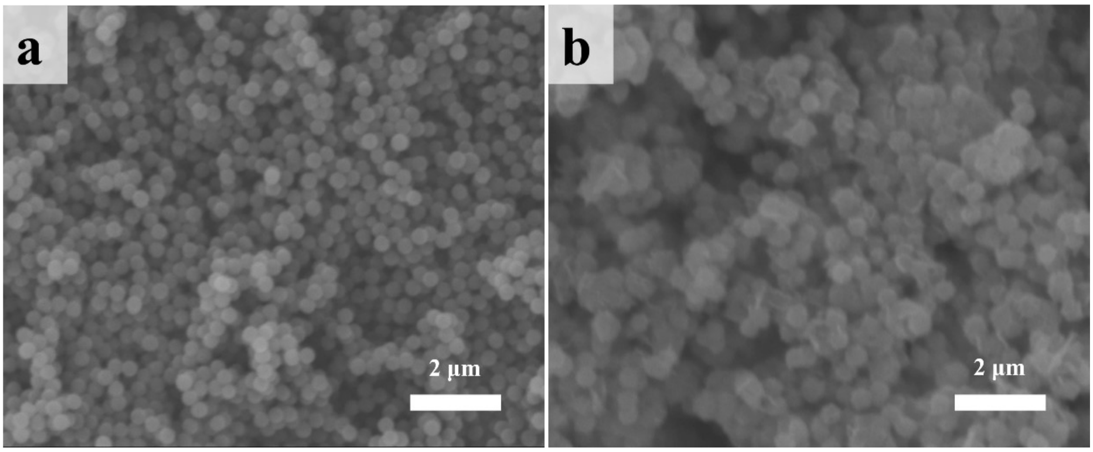

Fig. S2 SEM images of (a) SiO_2 and (b) SiO_2@CoO.

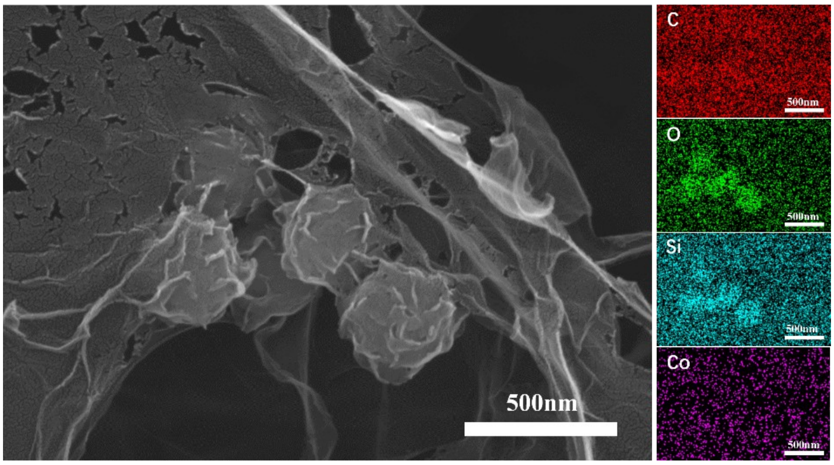

Fig. S3 EDS mapping images of SiO_2@CoO/GS.

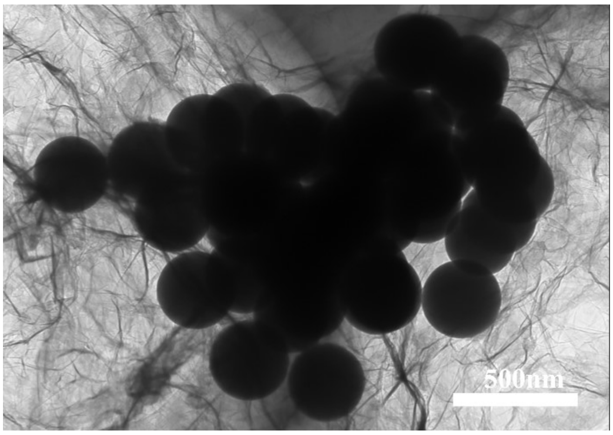

Fig. S4 TEM image of SiO_2/GS.

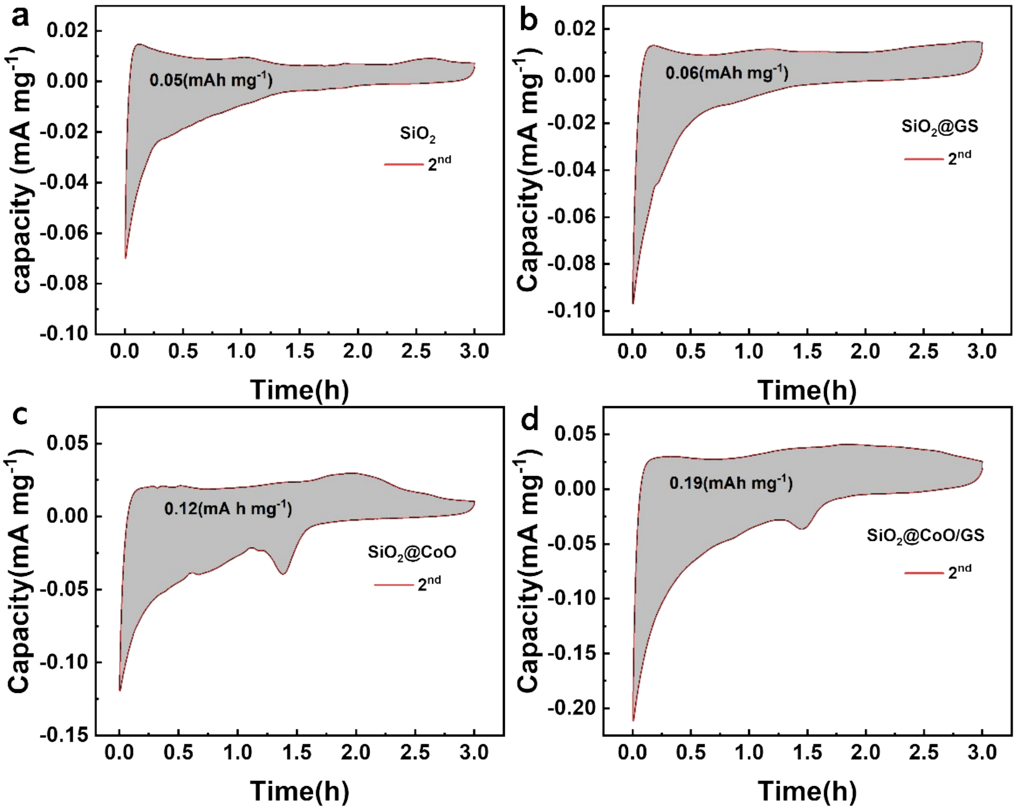

Fig. S5 Integrated area of the cyclic voltammetry curves for SiO_2 (a), SiO_2/GS (b), SiO_2@CoO (c) and SiO_2@CoO/GS (d).

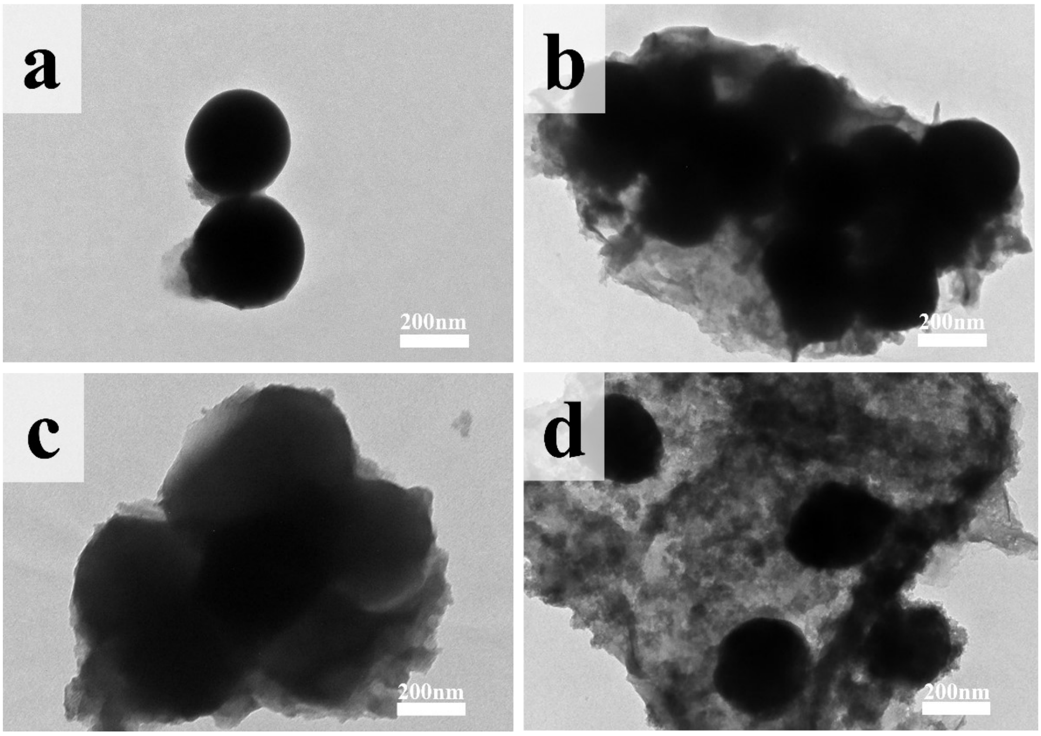

Fig. S6 TEM images of SiO_2 (a), SiO_2/GS (b), SiO_2@CoO (c) and SiO_2@CoO/GS (d) after 40 cycles.

Table S2 Comparison of the cycling and rate performance between this work and those reported in the literature.

Samples	Methods	Initial reversible capacity (mA h g^{-1}), Coulombic efficiency (%)	Performance	Current densities (mA g^{-1})	corresponding capacity (mA h g^{-1})	Capacity retention (%)
SiO_2@CoO/GS [this work]	solvothermal	764 at 200 mA g^{-1}, 47.2	Rate	100	707	100.0
				200	617	87.3
				400	543	76.8
				800	480	67.9
				1600	426	60.3
				3200	374	52.9
				6400	322	45.5
				12800	264	37.3
				100	866	122.5
			Cycling	200	738 after 500 cycles	113
SiO_2@rGo-Co[1]	hydrothermal and thermal treatment	310.2 at 100 mA g^{-1}, 57.1	Rate	100	310.4	100.0
				200	270.1	87.0
				500	198.2	55.6
				1000	154.8	49.9
				2000	112.7	36.3
				5000	70.4	22.7
				100	306.6	98.8
			Cycling	1000	144.9 after 2000 cycles	94.03
SiO_2/pC[2]	Sol-gel and hydrothermal	420 at 100 mA g^{-1}, 34	Rate	50	430	100.0
				100	390	90.7
				200	302	70.2
				400	209	48.6
				800	127	29.5
				1000	98	22.8
				50	417	97
			Cycling	100	625 after 600 cycles	49.9
SiO_2@C[3]	Sol-gel method	1110 at 10 mA g^{-1}, 47.5	Rate	100	437	100.0
				200	402	92.0
				400	350	80.1
				800	299	68.4
				1000	281	64.3
				2000	222	50.8
				200	410	93.8

			Cycling	100	511 after 100 cycles	73
NCSC[4]	Thermal treatment	565.4 at 100 mA g^{-1}, 53.65	Rate	100	530	100.0
				200	475	89.6
				500	410	77.4
				1000	380	71.7
				2000	345	65.1
				5000	273	51.5
				100	600	113.2
			Cycling	2000	254.6 after 500 cycles	77.0

References:

1. Q. An, X. Sun, Y. Na, S. Cai and C. Zheng, Chinese Chemical Letters, 2023, 34, 107305.

2. V. Gulavani, S. Kanade, A. Lokhande, M. Ottakam Thotiyl, B. John and A. Yengantiwar, *Energy Tech*, 2024, 2400094.

3. S. Ozen, O. Eroglu and N. Karatepe, *Nanotechnology*, 2023, 34, 485403.

4. J. Mao, M. Chen, Y. Deng, H. Liu, Z. Ju, Z. Xing and X. Cao, J Mater Sci, 2019, 54, 12767–12781.
